# *Pediatric Rheumatology *– a continuation of *Pediatric Rheumatology Online Journal*

**DOI:** 10.1186/1546-0096-5-1

**Published:** 2007-04-02

**Authors:** Alberto Martini, Charles H Spencer

**Affiliations:** 1 Professor Of Pediatrics, Ospedale Gaslini, Genova, Italy; 2 Professor of Pediatrics, University of Chicago, Chicago, Illinois, USA

## 

We welcome you to *Pediatric Rheumatology, now published by BioMed Central*. *Pediatric Rheumatology *is a continuation of the open access *Pediatric Rheumatology Online Journal*, launched in 2003 as the first solely pediatric rheumatology journal.

Our mission is to further research, scholarship, and education in pediatric rheumatology throughout the world and thereby improve the care of children with arthritis and rheumatic diseases. We hope to speed the growth of pediatric rheumatology in every country. We believe that a dedicated pediatric rheumatology journal has a better opportunity to help develop our field than existing, predominantly adult rheumatology journals. The journal is independent and international and is purposefully free of direct association with any one institution, organization, or commercial enterprise other than BioMed Central.

Our choice of the open access venue is intentional. We wish the pediatric rheumatology information in this journal to be freely available to anyone. We believe that this model is optimal for growth of pediatric rheumatology in both developed and developing countries [1,2]. Why is the online, open access venue preferable to the traditional subscription journals in rheumatology? First, all articles become freely and universally accessible online and so an author's work can be read by anyone at no cost. Articles, photos, figures, tables, and other components can be freely downloaded. Second, the authors, not the publishers, hold the copyright for their work and grant anyone the right to reproduce and disseminate the article, as long it is correctly cited and no errors introduced. Third, *Pediatric Rheumatology*'s articles will be archived in PubMed Central, the US National Library of Medicine's full-text repository of life science literature. These articles will also be archived in the repositories at the University of Potsdam in Germany, at INIST in France, the e-Depot, the National Library of the Netherlands' digital archive of all electronic publications, and in the new UK PubMed.

Why should we join the shift towards online, open access publishing? The traditional business model for scientific publishers requires restriction of access to published research so that the publishers can recoup the costs of publication. This restriction has deleterious effects, limiting full use of digital technology, and can be considered to be contrary to the interests of authors, funders, and the scientific and medical community as a whole. This traditional system is straining as increasing amounts of research are being published while library budgets have remained static. The open access approach treats publication as the last step in the research process:

1) The article processing charge (APC) covers the cost of publication so that there is free and immediate access to research and education articles.

2) The APCs ensure transparency and allow publishers to compete to provide the best service at the best price.

3) By linking the cost of publication to research budgets, APCs allow the publishing system to adjust to the ever-increasing volume of clinical and basic research [3].

*Pediatric Rheumatology *is open to submissions on all aspects of pediatric rheumatology, including: editorials; commentaries; research, both clinical and basic; clinical and basic science reviews; rheumatologic reviews for the generalist; significant case series or case reports; book reviews; letters to the editors; and drug studies. Submissions are online, reviews and decisions occur within four weeks in most cases, and publication online is rapid and continuous.

We hope that *Pediatric Rheumatology *will be useful to pediatricians, pediatric rheumatologists, adult rheumatologists, other professionals, and the lay public. Let's work together to help our kids with rheumatic diseases.

## Competing interests

Alberto Martini, MD and Charles H. Spencer, MD are Co-Editor-in-Chiefs of *Pediatric Rheumatology*.
